# mSpecs: a software tool for the administration and editing of mass spectral libraries in the field of metabolomics

**DOI:** 10.1186/1471-2105-10-229

**Published:** 2009-07-22

**Authors:** Bernhard Thielen, Stephanie Heinen, Dietmar Schomburg

**Affiliations:** 1 Institute of Biochemistry, University of Cologne, Cologne, Germany; 2 Max Planck Institute for Neurologic Research, Gleuelerstr. 50, Cologne, Germany; 3 Department of Bioinformatics and Biochemistry, Technical University of Braunschweig, Braunschweig, Germany; 4 Stieglitzweg 20, 50829 Cologne, Germany

## Abstract

**Background:**

Metabolome analysis with GC/MS has meanwhile been established as one of the "omics" techniques. Compound identification is done by comparison of the MS data with compound libraries. Mass spectral libraries in the field of metabolomics ought to connect the relevant mass traces of the metabolites to other relevant data, e.g. formulas, chemical structures, identification numbers to other databases etc. Since existing solutions are either commercial and therefore only available for certain instruments or not capable of storing such information, there is need to provide a software tool for the management of such data.

**Results:**

Here we present mSpecs, an open source software tool to manage mass spectral data in the field of metabolomics. It provides editing of mass spectra and virtually any associated information, automatic calculation of formulas and masses and is extensible by scripts. The graphical user interface is capable of common techniques such as copy/paste, undo/redo and drag and drop. It owns import and export filters for the major public file formats in order to provide compatibility to commercial instruments.

**Conclusion:**

mSpecs is a versatile tool for the management and editing of mass spectral libraries in the field of metabolomics. Beyond that it provides capabilities for the automatic management of libraries though its scripting functionality. mSpecs can be used on all major platforms and is licensed under the GNU General Public License and available at .

## Background

Metabolomics, the comprehensive analysis of metabolites present in a biological sample [1,2], is technically one of the most challenging fields in systems biology. While genetics has to handle the four digit code chemistry of the nucleic acids and proteomics the 20 letter code of amino acids [1,3], there are several thousands of metabolites with diverse organochemical properties known [2].

For the identification and quantification of metabolites a number of techniques have been available [4]. Besides nuclear magnetic resonance [5,6] and optical spectroscopies, e.g. Raman- and Fourier transform infra-red spectroscopy [7], a major part of the methods rely on chromatographic separation, either by gas chromatography, liquid chromatography or capillary electrophoresis, followed by a mass spectroscopic characterization of the substances [5]. During gas chromatography coupled to mass spectroscopy (GC/MS [8]) the boiling points of the compounds are usually decreased by derivatization prior to measurement in order to provide a higher yield in detection [4,9].

The detection of metabolites is typically accomplished by the comparison of obtained mass spectra and their retention time or retention index value with standards pooled in a library [9,10]. While this information may be sufficient for the identification, in many cases there is a need to add additional data to a library entry. One common task is e.g. to draw data attained from experiments onto metabolic pathway maps, and there are several tools to handle such maps, e.g. VANTED [11] and Cytoscape [12]. However, for the automatic mapping of the data it is necessary to connect a standard in a library to a metabolite on the pathway map, e.g. by utilizing its KEGG compound number [13].

For the maintaining of libraries there are several tools available, from which NIST MS Search [14] and AMDIS [15] are the most common ones. However, the possibilities to edit and manage mass spectra as well as associated information are limited. None of the programs is capable of handling more than two libraries at the same time, performing complex sort and filter options and automated operation via scriptable commands. Furthermore the data fields lack important areas such as multiple reference ions for quantification, KEGG compound numbers, InChI codes [16] or systems biology data like associated reactions, enzymes or genes. Therefore we present mSpecs, an open source based software for the manual and automated management of libraries used in chromatography/mass spectroscopy approaches.

## Implementation

mSpecs is released under the GNU General Public License [17] and was programmed using C++ and the Qt4-framework [18]. It can be compiled on all major platforms including Windows, Linux and MacOS X. An installer package for Windows platforms as well as documentation and source code is provided on the website of the project.

## Results

### Data fields

**Figure 1:**
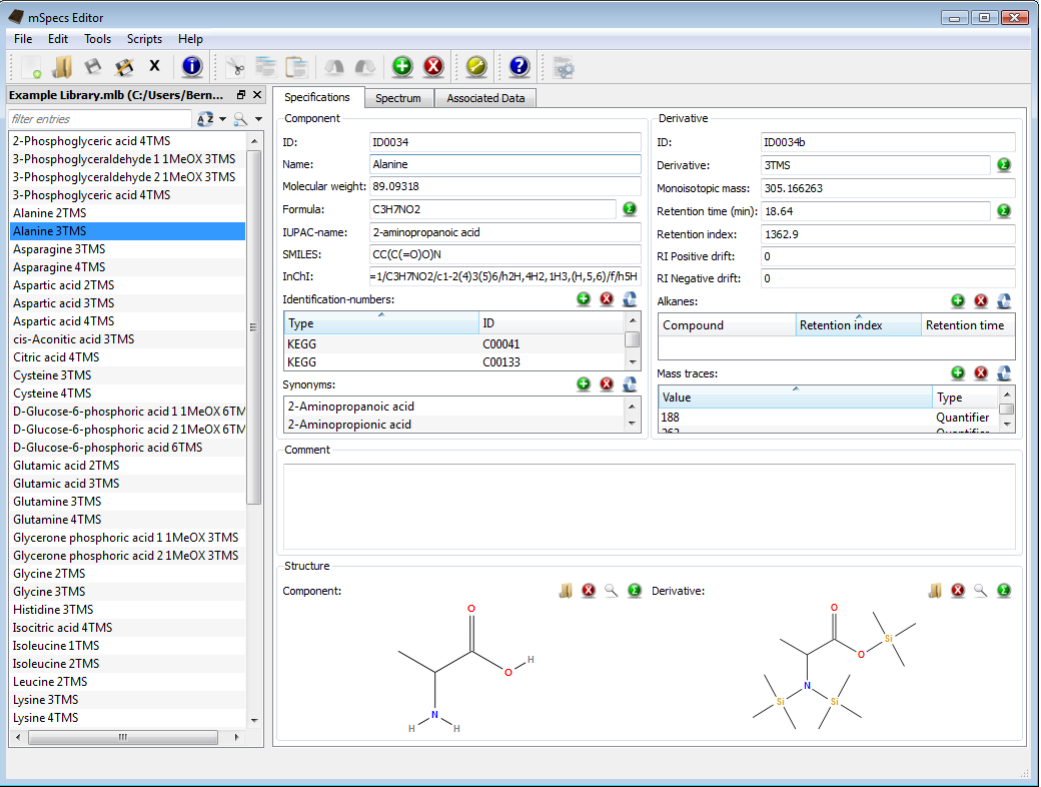
**A screenshot of the graphical user interface of mSpecs**. The specifications page showing a list of entries (left) and the data fields for the chosen compound (N, N, O-Tris-(trimethylsilyl)alanine; right).

mSpecs provides an easy-to-use graphical user interface (see figure 1). The workspace is divided into three pages (or tabs), on which data can be entered. The entries of the library are listed in a separate table on the left side, which can be undocked from the main window and moved to an arbitrary screen location. The given data fields cover all areas of interest, including viewing the spectrum in visual and tabular form, information such as retention time and Kováts retention index [10], identification numbers, e.g. from KEGG [13], ChemSpider [19] or HMDB [20] other chemical data like SMILES-codes [21], monoisotopic masses [22] and the author's name or the date of measurement. A complete overview can be found in table 1. Since in the field of GC/MS it is often necessary to maintain the information of two chemical entities, the metabolite and its derivative, data fields for both substances are provided.

**Table 1:** Available data fields, their type and whether they are sortable or filterable.

**Field**	**Type**	**Comment**	**Sortable/Filterable**
*Specifications Component*			
ID	string	identification number	yes/yes
Name	string	identifier	yes/yes
Molecular weight	double	molecular weight	yes/yes
Formula	string	chemical formula	no/yes
IUPAC-name	string	IUPAC-name	no/yes
SMILES	string	SMILES-code [21]	no/yes
InChI	string	InChI-code [16]	no/yes
Identification numbers	2D array^1)^	e.g. KEGG compound number [13]	no/yes
Synonyms	array of strings	synonyms	no/yes
Derivatives			
ID	string	identification number	yes/yes
Derivative	string	identifier	yes/yes
Monoisotopic mass	double	monoisotopic mass [22]	yes/yes
Retention time (min)	double	Retention time	yes/yes
Retention index	double	Kováts retention index [10]	yes/yes
RI positive drift	double	Positive drift of retention index	yes/yes
RI negative drift	double	Negative drift of retention index	yes/yes
Alkanes	2D array^1)^	reference list of alkanes	no/no
Mass traces	array of doubles	list of reference ions for quantification	no/yes
Comment			
Comment	string	commentaries	no/yes
Structure			
Component	molecule^2)^	chemical structure of the component	no/no
Derivative	molecule^2)^	chemical structure of the derivative	no/no

*Spectrum*			
Visual representation	--	graphical representation	no/no
Tabular representation	2D array^1)^	tabular representation	no/no

*Associated data Measurement*			
Author	string	author of the measurement	no/yes
Date	date	date of measurement	yes/yes
Device	string	device used	no/yes
Method	string	explanation to the method	no/yes
Column	string	used chromatographic column	no/yes
Experimental conditions	string	further experimental conditions	no/yes
Systems Biology Data			
Reactions	array of strings	associated reactions	no/yes
Enzymes	2D array^1)^	associated enzymes	no/yes
Genes	2D array^1)^	associated genes	no/yes

^1) ^Table with a given number of columns and arbitrary number of rows.

^2) ^Object storing a molecular structure.

### Capabilities of the user interface

Each of the data fields can be activated or deactivated using the built-in settings dialog and the user interface will dynamically fit to the available space. This way, the scientist is able to adapt the interface to his needs without losing data in the hidden fields.

There is no limit to the number of open files and the user is able to copy and paste or drag and drop entries between the libraries. Any user input can generally be undone by the program's undo/redo functionalities. In order to navigate through the list of entries, there are multiple sort and filter options available (see table 1). The maximum number of simultaneously maintained entries depends only on the size of the main memory of the computer. Assuming one gigabyte of free memory and an average size of an entry from four to 40 kilobytes, there are 25,000 to 250,000 entries that can be maintained simultaneously. Bearing in mind that the NIST MS library [14], one of the largest mass spectral libraries available, contains about 191,000 spectra, this should be enough for most of the tasks.

Much of the data is entered in simple text boxes. However, certain information cannot be stored in simple text strings, so that a special treatment is applied. An example is the graphical representation of the structures of metabolite and derivative. Structures can be imported in MDL mol file [23] or CML [24] version 1 or 2 format and can be displayed. Furthermore structures can be exported in MDL mol file, CML version 1 and 2, scalable vector graphics [25] and several image formats such as jpeg.

It is possible to compute the molecular formula from the structure, the molecular weight of the component or the monoisotopic mass of the derivative based on the formula and furthermore the Kováts retention index [10] starting from the retention time and a reference list of alkanes. Moreover certain fields like the identification numbers or the fields in the systems biology area (see table 1) provide a link to corresponding information on the internet.

### Loading and saving of libraries

mSpecs provides various import and export options. In Addition to its own binary format, which is optimized for fast disk operations while maintaining small size, mSpecs is able to load and save the AMDIS/NIST mass spectral format [15], which is also supported by the Xcalibur software package from Thermo Scientific [26]. As a second file format JCAMP-DX [27] is supported, which again can be used together with the ChemStation software by Agilent Technologies [28].

A major part of the data fields can be exported into a tab-delimited text file, which then can be viewed in spreadsheet software. An export into the portable document format (pdf) [29] as well as printing is also possible. Furthermore mSpecs provides an implementation of the extensible markup language (xml) [30], which serves as an interexchange format for prospective developments.

### Automation using scripts

In order to allow automated manipulations of the library a scripting language based on the ECMAScript scripting language [31], which is also the basis of e.g. JavaScript, was implemented. Within the scripting environment it is possible to access all data fields and the calculations and supporting functions such as disk input/output and user interactions. Additionally the scripts are embedded into the undo/redo framework of mSpecs. The use of scripts can considerably simplify the maintenance of large libraries. Below a script is listed that calculates the chemical formula from the given structure of each compound in a library. This script requires less than two seconds on a library with more than 500 entries on a 2 GHz processor.

/* description:

This is a demo script to illustrate the automated operation of mSpecs.

*/

// process every entry in the active library.

for (var i = 0; i < library.length; ++i)

{

   // get structure of the actual component.

   var molecule = library.at(i).moleculeComponent;

   // calculate chemical formula from the structure.

   var formula = tools.calculateFormulaFromStructure(molecule);

   // store formula in the corresponding data field.

   library.at(i).formula = formula;

}

### Future development

Our primary goal is to provide further interoperability with other tools like MetaQuant [32] or Bioclypse [33] in order to make mSpecs usable for a larger community. The data fields, the user interface and the scripting functionalities will be extended on the basis of user feedback. More vendor-specific file formats will be supported depending on available implementation details. We are currently working on a suite to view and analyze data obtained from GC/MS or LC/MS-experiments similar to AMDIS [34], but with more possibilities such as handling high-resolution mass spectroscopic data. mSpecs will be part of this suite as a library maintaining tool.

## Discussion and conclusion

mSpecs is a versatile tool for the management and editing of mass spectral libraries in the field of metabolomics. Beyond that it provides capabilities for the automatic management of libraries though its scripting functionality. mSpecs can be used on all major platforms and is licensed under the GNU General Public License and available at 

## Availability and requirements

Project name: mSpecs;

Project home page: ;

Operating system(s): platform independent;

Programming language: C++; Other requirements: Qt 4.4 (or higher);

License: GNU GPL

## Abbreviations

GC/MS: gas chromatography – mass spectrometry; LC/MS: liquid chromatography – mass spectrometry.

## Authors' contributions

BT carried out the major part of the program design and did the major part of the programming. SH participated in the design and testing of the program. DS consulted and supervised the project. All authors read and approved the final manuscript.
